# Quadrupedal water launch capability demonstrated in small Late Jurassic pterosaurs

**DOI:** 10.1038/s41598-022-10507-2

**Published:** 2022-04-21

**Authors:** Michael Pittman, Thomas G. Kaye, Hebert B. Campos, Michael B. Habib

**Affiliations:** 1 School of Life Sciences, The Chinese University of Hong Kong, Shatin, Hong Kong SAR China; 2 Department of Earth Sciences, University College London, London, WC1E 6BT UK; 3 Foundation for Scientific Advancement, Sierra Vista, Arizona, 85650 USA; 4 Independent Researcher, Campina Grande, Paraíba, Brazil; 5 Dinosaur Institute, Natural History Museum of Los Angeles County, Los Angeles, CA 90007 USA

**Keywords:** Evolution, Palaeontology

## Abstract

Pterosaurs thrived in and around water for 160 + million years but their take-off from water is poorly understood. A purportedly low floating position and forward centre of gravity barred pterosaurs from a bird-like bipedal running launch. Quadrupedal water launch similar to extant water-feeding birds and bats has been proposed for the largest pterosaurs, such as *Anhanguera* and *Quetzalcoatlus*. However, quadrupedal water launch has never been demonstrated in smaller pterosaurs, including those living around the Tethys Sea in the Late Jurassic Solnhofen Lagoon. Using Laser-Stimulated Fluorescence, we singled out aurorazhdarchid specimen MB.R.3531 that alone preserved specific soft tissues among more than a dozen well-preserved Solnhofen pterosaur specimens. These soft tissues pertain to primary propulsive contact surfaces needed for quadrupedal water launch (pedal webbing and soft tissues from an articulated forelimb) that permit robust calculations of its dynamic feasibility without the need to make assumptions about contact areas. A first-principles-based dynamics model of MB.R.3531 reveals that quadrupedal water launch was theoretically feasible and that webbed feet significantly impacted launch performance. Three key factors limiting water launch performance in all pterosaurs are identified, providing a foundation for understanding water launch evolution: available propulsive contact area, forelimb extension range and forelimb extension power about the shoulder.

## Introduction

Pterosaurs repeatedly invaded marine habitats from their traditionally terrestrial realm, but the details of this transition are unclear. Based on their estimated low floating posture^1^, pterosaurs would have been susceptible to drowning if they spent prolonged time on the water’s surface e.g., following a dive or accidental submersion. As pterosaurs habitually fed in or around the water, it is not unreasonable to assume that some species would have been capable of effective water launches. Most living water birds use a dynamic running launch, but this mode was out of reach for pterosaurs owing to their purportedly low floating position and forward centre of gravity^1^. A diverse group of water-feeding living birds and bats utilise alternative wing-dominated quadrupedal water launches, which is a category of water launch that pterosaurs should be capable of. For example, fishing bats in the genus *Noctilio* are able to launch from water using their wings to push free of the surface^2,3^. Quadrupedal launch has also been suggested as the mode adopted by pterosaurs from terrestrial substrates^4,5^. Habib and Cunningham^6^ demonstrated the dynamic capacity of quadrupedal water launch in large pterosaurs using the Cretaceous pterodactyloids *Anhanguera* and *Quetzalcoatlus.* They also proposed a range of anatomical specialisations in their skeletons that would be expected for this behaviour, especially in the pectoral girdle. However, this analysis was limited in scope and reported in an extended conference abstract so did not investigate the parameter sensitivity in detail. Thus, significant details of the nature of pterosaur water launch remain elusive. This study reports an expanded and revised version of the water launch model created by Habib and Cunningham^6^, and then uses this model to consider the potential for water launch in small pterosaurs, using the historic specimen MB.R.353 as an exemplar. Smaller pterosaurs would be expected to be less power-limited than the largest species, simply on account of mass-specific power scaling. However, the surface tension effects would be comparatively greater for small pterosaurs, and this could make water launch prohibitive for some small pterosaurs.

**Figure 1:**
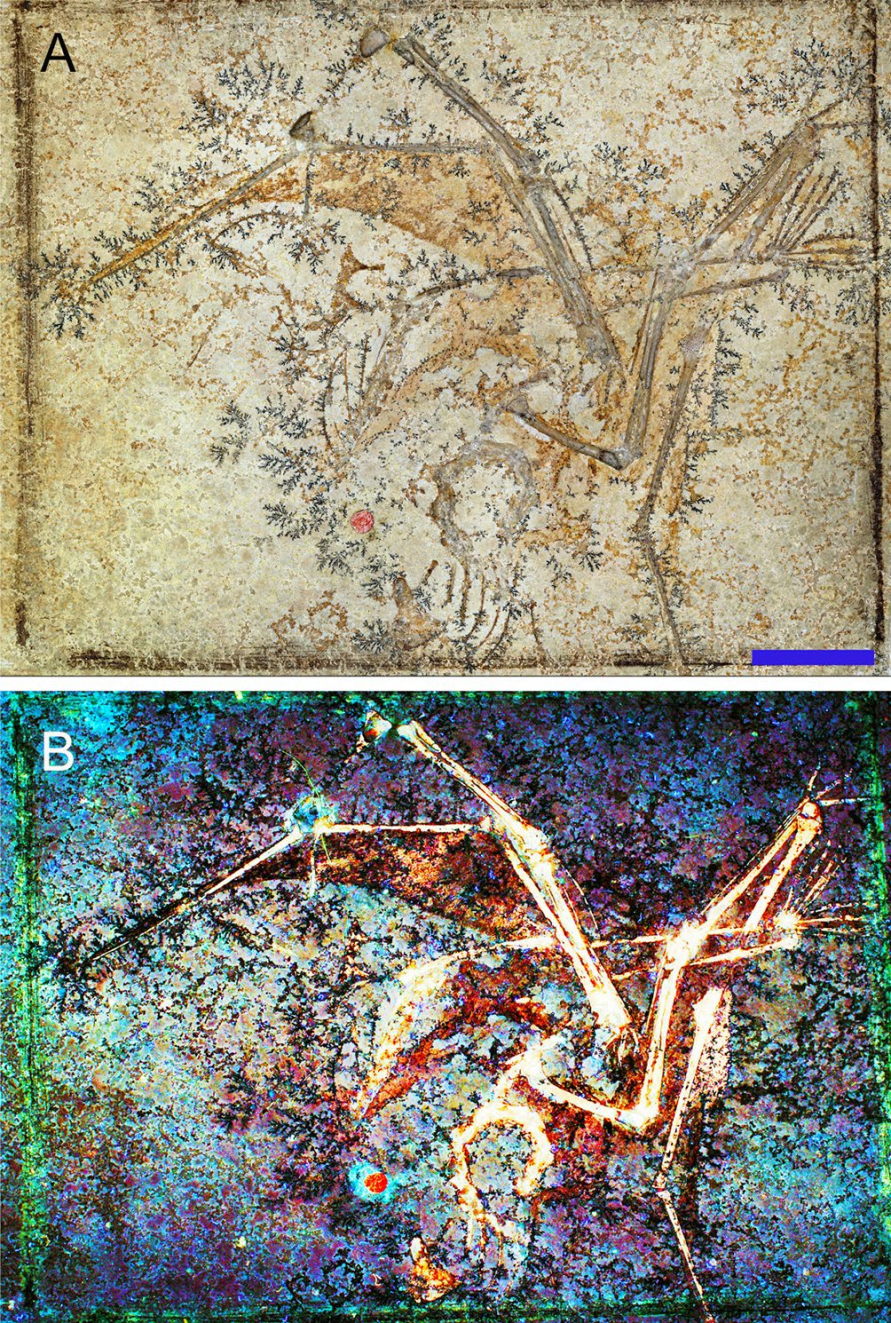
Skeleton and associated soft tissues of the aurorazhdarchid pterosaur specimen MB.R.3531a. (**a**) White light image of slab. Scale = 20 mm. (**b**) LSF image of slab. Soft tissues visible under white light are clearer under LSF, whilst speculative soft tissues under white light are confirmed by the technique. Otherwise invisible soft tissues are also revealed under LSF. See measurements in .xlsx file in the Supplementary Information S1.

Using Laser-Stimulated Fluorescence, a historic aurorazhdarchid specimen MB.R.353 was singled out from more than a dozen small well-preserved pterosaurs that originally lived in the Late Jurassic Solnhofen Lagoon on the fringes of the Tethys Sea (see ‘Solnhofen Pterosaur Fossils’ tab in Supplementary Information S1). The wing is preserved in a mostly folded, articulated state with intervening soft tissues preserved (Fig. 1). The feet of the specimen are preserved in an open position and pedal webbing is present (Fig. 1). These positions are similar to what was proposed by Habib and Cunningham^6^ for the water contact positions of the limbs during quadrupedal water launch in the large pterosaurs *Anhanguera* and *Quetzalcoatlus.* This enabled robust estimates of quadrupedal water launch performance of MB.R.3531 using measured propulsive contact areas, rather than assumed areas. The specimen’s pedal webbing also permits specific sensitivity tests of how pedal webbing area affects quadrupedal water launch performance. Thus, MB.R.3531 is the ideal specimen for evaluating water launch feasibility and performance in small pterosaurs.

MB.R.3531 consists of a rectangular slab (MB.R.3531a) and counterslab (MB.R.3531b) measuring ~ 159 mm by ~ 122 mm that is housed at the Museum für Naturkunde Berlin, Germany (Fig. 1). The main slab preserves most of the skeleton, including most of the fossilised soft tissue. The skeleton is represented by almost complete forelimbs, nearly complete hindlimbs as well as cervical and dorsal vertebrae and some ribs. MB.R.3531 preserves the fossilised wing membrane, which are preserved as dark stains and impressions in other contemporaneous specimens^7,8^. MB.R.3531 is provisionally assigned to *Aurorazhdarcho* (Pterodactyloidea: Aurorazhdarchidae) based on shared features with other *Aurorazhdarcho* specimens^9^.

## Results

### Basic description of soft tissues

Pterosaur specimens preserving fossilised soft tissue are extremely rare. To our knowledge there are ~ 30 specimens with preserved wing membranes and five others with evidence of webbed feet^10–14^. Therefore, in addition to obtaining the pedal area measurements for water launch analysis, we first provide a review of the soft tissues in MB.R.3531, since LSF highlighted features that were difficult to discern under white light conditions.

**Figure 2:**
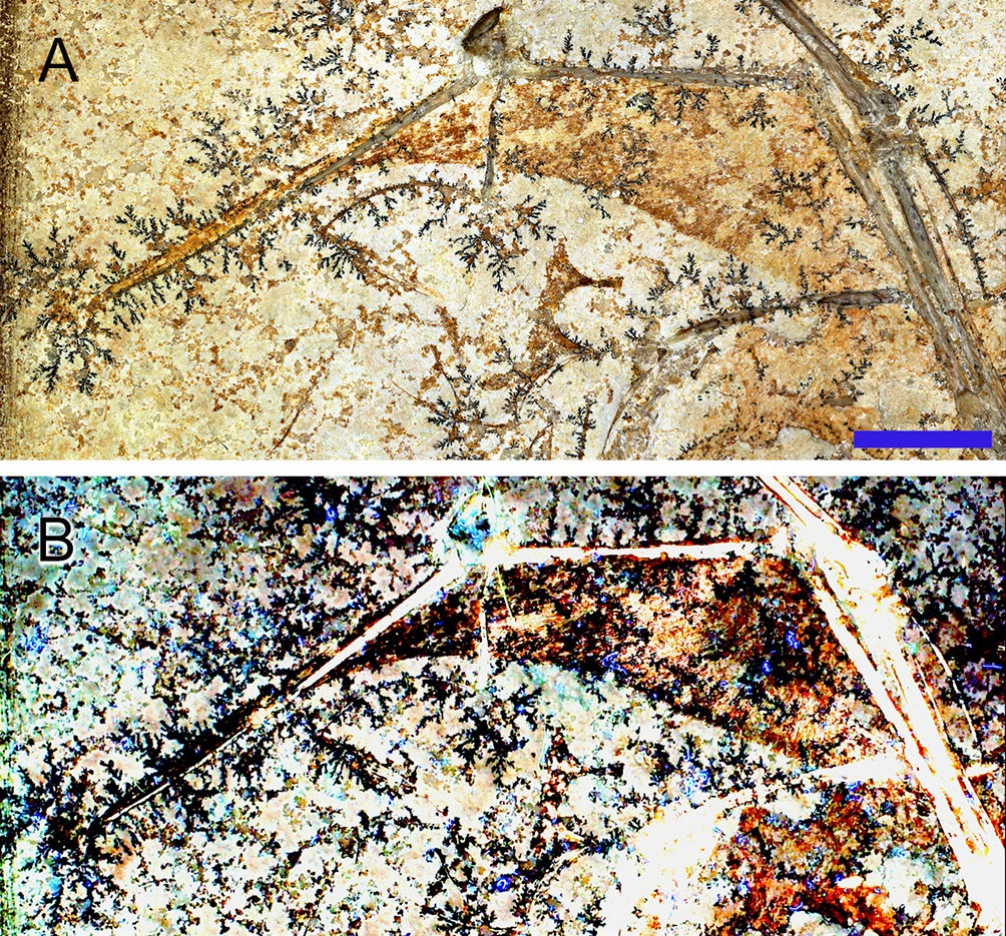
Left wing of aurorazhdarchid pterosaur specimen MB.R.3531a. (**A**) White light image. Scale = 15 mm. (**B**) LSF image defines details of the actinofibril complex and leading edge of the wing. Brown coloured portions of the anterior part of the wing appear to be preserved remnants of the retrophalangeal wedge.

The wing membranes of MB.R.3531 are best preserved in the left wing, where the actinopatagium (we use this term herein, but this is also known as the dactylopatagium) lacks any noticeable imperfections and is folded over at its distal tip (Fig. 1). The right-wing membrane is continuously preserved along the full length of the arm, but the folded position obscures details (Fig. 1). Under LSF, brown-coloured anterior portions of the left actinopatagium appear to be preserved parts of the retrophalangeal wedge that have a distal margin subparallel to the trailing edge of the wing (Fig. 2). According to Monninger, Frey and Tischlinger^15^, the retrophalangeal wedge is a supporting structure along the caudal face of the wing finger that likely consisted of fibrous tissue e.g. interwoven tensoelastic elastin or high tensile collagen fibres. The actinofibrils of the left actinopatagium are near perpendicular to the first phalanx of digit IV but are subparallel to the remaining distal phalanges (Fig. 2). Compared to other taxa, the actinofibrils transition from strongly span-wise to chord-wise relatively abruptly and more distally, near the second interphalangeal joint rather than closer to the wrist (Fig. 2). As preserved, the wing had a narrow chord distal to the wing “pivot” (fourth metacarpophalangeal joint), especially when considering extension of the wing in flight, which would stretch the membrane span-wise. The proximal chord cannot be determined from the specimen.

**Figure 3:**
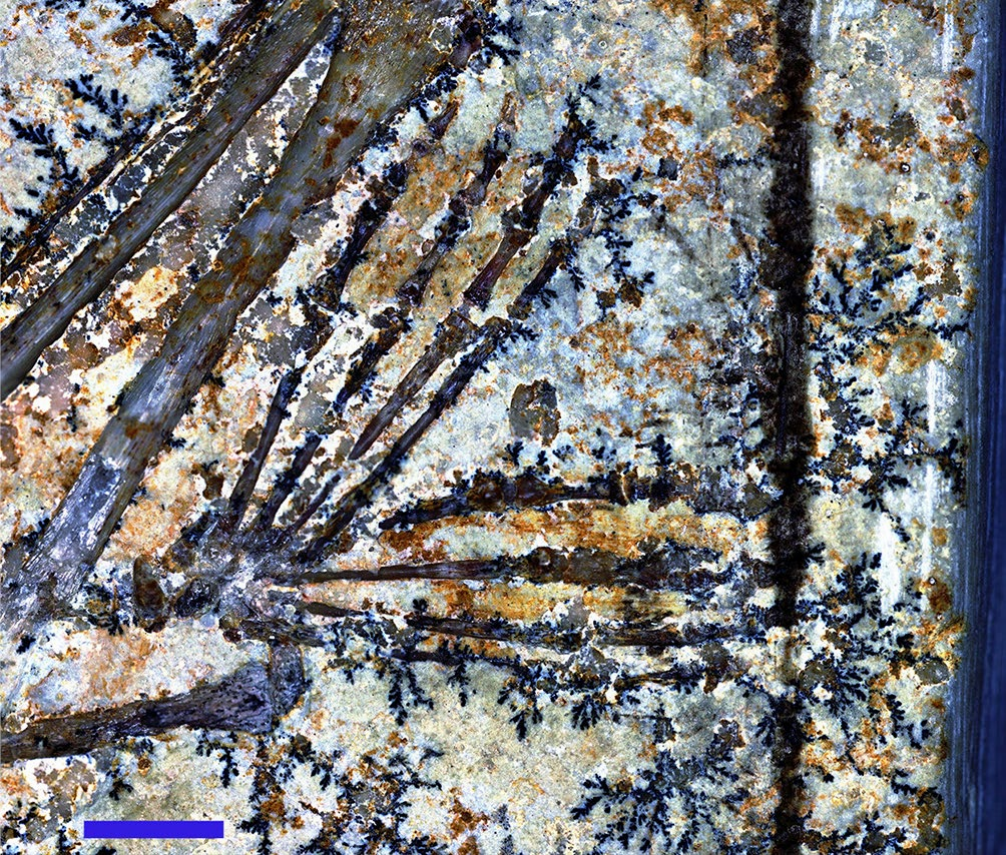
Webbed feet in the aurorazhdarchid pterosaur specimen MB.R.3531a. Parallel actinofibral-like fibres extend from the base of the toes to the base of the claws. Enhanced white light image. Scale = 4 mm.

The feet of MB.R.3531 are totipalmate (all four digits are entirely joined by webbing) (Fig. 3), resembling the condition in many living seabirds such as pelicans and gannets^16^. Webbed feet are preserved in at least two other pterodactyloids from the Solnhofen Limestone. In *Pterodactylus* JME SOS 4784, parallel-oriented fibres are visible between digits I and II of the right pes^10,11^. In *Rhamphorhynchus muensteri* JME SOS 4785, there is preserved webbing between metatarsals I-IV and digits I-IV of the right pes^11,17^. Under UV, suspected phosphatised fibres are visible between some digits of the pes^11^. In both specimens, the pedal webbing extends longitudinally to the base of the claws and the fibres appear similar in morphology to the actinofibrils of the pterosaur wing. This is also observed under white light and LSF in MB.R.3531 (Fig. 3). Confirmation that the webbing contained fibrous reinforcement further supports the idea that the webbed feet could be used along with the wings as propulsive surfaces during water launch.

### Water launch modelling

To push against the water surface with the wings, pterosaurs would need to slightly internally rotate the wings and push with a mostly folded wing by retracting it at the shoulder. That range of shoulder rotation cannot be tested in the present specimen, as the shoulder joint is incompletely preserved. However, it is conservative to assume that pterosaurs had such a range of motion given that it is observed in all powered flyers, living crocodilians and birds. The initial area of contact to escape the water’s surface tension would include the area of the folded wings and both webbed feet. After this initial push, additional propulsive phases across the water surface could occur by pushing with a smaller portion of the wing near the “wing pivot” joint (i.e., the fourth metacarpal to wing finger joint). This would constitute a form of drag-based propulsion, with the inserted portion of the wing essentially sculling in the water. This specific mallard-like retraction-based kinematic is suggested by the low estimated floating posture of pterosaurs^1^ and the notably large wing retraction muscles at the shoulder of marine pterosaurs such as *Anhanguera*^6^.

The dynamics-based approach used here takes onboard varying estimates of available power and limb speeds, while applying measured values for contact area and limb segment lengths. Crucially, this approach avoids the need to adopt specific kinematics that are currently difficult to constrain due to uncertainties in the specifics of the low pterosaur floating posture and their range of motion. However, a more conservative and broader assumption was made that the wings could be sufficiently positioned or rotated to push against the surface of the water with a substantial percentage of their folded area. From this starting point, we examine the potential sensitivity of water launch performance with the variables of contact area, range of motion, and limb lengths. Thanks to the fluorescence of the potential propulsive contact surfaces under LSF imaging, we can use *measured* values for contact area, making the estimates of variable sensitivity in other parameters better constrained. We assumed body proportions similar to *Pterodactylus* for the unknown parts of the animal (trunk and skull), which enable an analysis that expands insights of quadrupedal water launch to smaller pterosaurs from aquatic settings.

Water take-off capacity was broadly estimated in MB.R.3531 using a dynamics model that makes limited (and conservative) assumptions regarding kinematics. To that end, it is designed specifically to determine performance *only*, and it should not be used to attempt to deduce exactly how a water launch would look. The complete model is provided in the ‘Quadrupedal Water Launch Model’ tab of the .xlsx file in the Supplementary Information S1. See “Water take-off estimation” section in the “Materials and methods” for further information. The general patterns of performance, particularly predictions regarding sensitivity to inputs, are the primary data output of this modelling exercise. Under the most conservative model values, calculations estimate that MB.R.3531 was capable of taking off from the water surface with a single escape push or with 1–2 follow-up bounding phases. The added propulsive area of the pedal webbing has a notable effect on overall launch performance, increasing the estimated propulsive accelerations by 20% and reducing the required number of propulsive bounding phases. The results of this model therefore predict that MB.R.3531 was capable of water launch, even with conservative input values (from living animals on the lower end of their power output and on the higher end of drag coefficients for semi-aquatic forms of similar size). The model predicts that water launch performance in pterosaurs would be particularly sensitive to three factors: available propulsive contact area, forelimb extension range and forelimb extension power about the shoulder.

## Discussion

### Implications of wing soft tissue details for aeroelasticity

We note that MB.R.3531 provides useful new information about the functional anatomy of pterosaur wings, particularly the structure of the wing near the base of the wing finger. Actinofibril size, orientation, and density in the preserved wing membrane can be roughly deduced from white light photography, but these features are more precise under LSF. Actinofibrils are regarded as epidermal, keratinous structures^18,19^ located under the surface of pterosaur patagia^20^. They likely originated from modified epidermal scales that formed in place as part of the dorsal epidermis of the actinopatagium^8^. Actinofibrils are found throughout Pterosauria, including in early-diverging pterosaurs from the Norian stage of the Upper Triassic^21,22^. Given this phylogenetic breadth and early appearance, actinofibrils are likely a plesiomorphic feature of pterosaurs. In MB.R.3531, the complete series of actinofibrils—or actinofibrillar complex^23^—is preserved in the actinopatagium.

Metacarpals (MC) I-III reach nearly to the carpus. They have shifted distally, as a group, past the wing pivot joint. This is consistent with the hypothesis that MC I-III translated distally when in walking position and would push the fingers beyond the wing pivot from which they could then flex under the pivot to support walking^24^, though it is not conclusive. The in situ preservation of the metacarpals demonstrates that the palmar portion of the wingspan had a broader soft tissue attachment area (and was deeper in the chord wise direction) than previously appreciated.

The pteroid is extremely thin and elongate. If it were in life position against the medial aspect of the pre-axial carpal^25^ then it would reach close to the elbow, suggesting that about 50% or more of the leading edge of the propatagium was reinforced by this bony structure in flight. This would have had direct impact on the ability to take up chordwise slack from the wing (more span reduction was possible than some other taxa) and on leading-edge stiffness and resistance to aeroelastic flutter. The ability to effectively control aeroelastic flutter is particularly important for performance of a high lift wing, which will occur either at a large angle of attack or high airspeed^26^, but would have been modulated by a muscular wing root fairing^27^. The minimum speed required for launch is highly sensitive to the maximum lift coefficient. As a result, control of aeroelastic flutter at low speeds (i.e., at high angles of attack) is predicted to be particularly important for launch and slow soaring (e.g., thermal soaring). At intermediate speeds, control of aeroelastic flutter is proportionally less constraining. However, in rapid flight (e.g., dynamic soaring), aeroelastic control again becomes potentially performance limiting, since a compliant wing can flutter at high speeds even when the angle of attack is low.

Palmer used a structural model to estimate the membrane tension loading in pterodactyloids^19^. He proposed that the strain is subparallel to the mediolateral axis of the wing and is constant across the anteroposterior width of the membrane, supporting all the wing phalanges. For the wing membrane to support flight loads he found it must not stretch excessively to retain an efficient aerodynamic shape, independent of actinofibril orientation. Palmer (2017) concluded that the actinofibrils would mostly load in tension, preventing excessive aeroelastic flutter when the wing membrane became cambered under aerodynamic load. Bennett proposed that the main function of the actinofibrils is to prevent narrowing of the wing membrane under tension and redirecting the spanwise tension to the proximal phalanges, reducing loads on the distal phalanges^18^. The distribution of the actinofibrils in the actinopatagium of MB.R.3531 varies along the wing membrane (Fig. 2). Of particular interest in MB.R.3531 is the sudden transition in actinofibril orientation just behind the proximal interphalangeal (PIP) joint from largely spanwise to mostly chordwise. The moderate packing and partially chordwise orientation of the actinofibrillar complex around the PIP joint in MB.R.3531 is consistent with the model proposed by Palmer^19^, in which the actinofibrils load in tension to control aeroelastic instability of the wing. However, we also note that the wing is preserved in a partially folded position. At full wing extension, the transition zone from spanwise to chordwise actinofibril orientation would shift inboard, and the spanwise fibrils would become more tightly packed. This estimated in-flight configuration for the actinofibril orientation in MB.R.3531 appears to be compatible with the model of Bennett^18^ (though a more rigorous analysis of compressive load response is needed to say more). We suggest that both models may be correct, with the actinofibrils serving multiple functions over different phases of flight. Specifically, we suggest that the actinofibrils were critical to preventing aeroelastic flutter during span changes (as per^19^), as well as during wing opening immediately after launch. During cruising flight, the actinofibrils could continue to play a role in maintaining tension and shape, but the shift in the chordwise transition zone to a more proximal location, along with the tighter fibril packing, might have allowed the actinofibrils to then assist in redirecting spanwise tension to the proximal portion of the proximal phalanx and the fourth metacarpal (consistent with^18^). We recommend that other pterosaurs with well-preserved wing membranes also be re-examined with this potential for mixed functionality in mind. Variations between taxa could help to elucidate comparative differences in flight speeds, capacity for span reduction, and flight efficiency.

### Water launch and paleoecology

The Solnhofen pterosaur fauna is particularly interesting for investigating water launch in a comparative context, since both pterodactyloid and non-pterodactyloid pterosaurs of similar size and wingspan were living sympatrically. Our analysis of MB.R.3531 highlights the importance of the propulsive contact areas in the forelimbs and hindlimbs for water launch. If the wings were used in launch from a fully or partially folded position, then we can deduce that the expanded metacarpal of pterodactyloids (like MB.R.3531) would provide an advantage by increasing both the relevant contact surface area as well as the folded forelimb length (thereby increasing the effective stroke length of the forelimbs). Non-pterodactyloid Solnhofen taxa such as *Rhamphorhynchus* bear a basal condition of a short metacarpal IV, which might have made them less efficient at water launch. While we have no evidence at present to indicate that this would *prevent* rhamphorhynchids from water launching, it might have affected their niche space and/or ecology (e.g., rhamphorhynchids may have been less likely to use plunge diving or other feeding methods that require habitual entry to the water).

The results of this study suggest that a quadrupedal water launch was dynamically feasible for MB.R.3531. Our analysis of MB.R.3531 provides important insights regarding variables that would have a disproportionate effect on water launch performance, confirms that pedal webbing could be important in this regard, and creates a comparative context for further investigations of water launch potential and evolution in pterosaurs. While many small pterosaurs likely had enough contact area, range of motion, and power to escape the water surface, it is quite plausible that more terrestrial (i.e., less marine-adapted) taxa may have been unable to water launch, especially if a lack of pedal webbing limited propulsive contact area. Further analyses of additional specimens will be required to address this in the future.

This model assumes very simple arcs of motion from both the forelimbs and hind limbs through highly conservative ranges of motion. The assumed ranges of motion can be found in the associated spreadsheet as angles that are used to calculate arc lengths (See Supplementary Information S1). We took this approach because the ranges of motion of pterosaur joints are still largely unknown and somewhat contentious^28^. It is likely that the true ranges of motion exceeded what we have built into our model, and this could significantly improve performance. Other authors using our model are encouraged to use any range of motion (ROM) data they may have to adjust these sweep angle parameters. We also note here that our model assumes a tightly folded wing for the forelimb propulsive surface. This assumption is based upon the observation that opening the wing, while it provides some additional surface for propulsion, adds a disproportionate amount of surface tension for a small pterosaur. This constraint is therefore considered robust for a small pterosaur. The wing could, however, be opened more for additional propulsive area in a larger pterosaur, given the non-linear reduction in relative surface tension forces with size.

Energetically efficient movement out of the water would have provided a significant foraging advantage as this would make habitual feeding from the water surface far less risky by minimising the risk of drowning. Our results reinforce the idea that at least some pterosaurs spent significant parts of their life in and around water^5,29,30^. Future work that can refine calculations of quadrupedal water launch by including direct measurements of range of motion and muscle volume information would be invaluable. Priority should be given to sample even larger collections of specimens to try and identify a diversity of specimens preserving propulsive contact surfaces so that the origin and refinement of quadrupedal water launch capabilities can begin to be investigated.

## Materials and methods

### Laser-stimulated fluorescence (LSF) imaging

LSF imaging was performed with a custom-built laser system following the augment protocol of Wang et al.^31^ based on the original by Kaye et al.^32^. A violet laser diode (0.60 W 405 nm) was projected into a laser line using a line lens (Laser Line Optics, Canada). This laser line was mechanically scanned back and forth over the specimen in a dark room. A Nikon D810 camera fitted with a laser blocking filter recorded the laser-stimulated fluorescence produced by the fossil during the exposure. The images were post-processed in *Photoshop CS6* for colour equalisation and colour balance. UV imaging was performed using a portable UV lamp (Raytech) in two different intensity phases to generate distinct contrasts (LW-365 nm and SW-254 nm). UV photographs and corresponding white light ones were taken with a Canon EOS 80D camera. The UV images did not produce any results that were not already visible under LSF. A high-resolution panorama reconstruction was produced using macrophotographs processed with the software *Hugin 2016* version 2.0. Linear measurements and angles were taken digitally from our high-resolution images as these were photographed in-plane. This involved using the software *ImageJ* version 1.47, *Screen Calipers* and *Screen Protractor* version 4.0.

### Water take-off estimation

Water take-off capacity was broadly estimated in MB.R.3531 using the model and measurements provided in the .xlsx file in the Supplementary Information S1. The model equations are based on first principles and known scaling relationships in living flying animals. The focus was on a dynamics perspective, rather than attempting to reconstruct a specific kinematic for water take-off. The model primarily works by breaking the launch cycle into discretised time intervals. This includes an initial escape phase and a propulsive bound sequence, the latter being split into a series of time intervals of its own. A power balance is solved for each time interval, with residual power adding to launch acceleration. When the sum of accelerations reaches the estimated minimum launch velocity, then the launch is successful. If a series produces zero or negative power residuals, it indicates that launch is not possible with the input values given.

The primary kinematic assumptions are that the forelimbs and hind limbs were both submerged (or could be submerged) immediately prior to take-off (sensu^1^) and that both the hindlimbs and forelimbs were capable of a power stroke in the parasagittal plane. The latest range of motion estimates for the pterosaur hind limb^28^ suggest that they had more limited abduction than previously modelled and operated primarily in a vertical plane. As a result, the use of the uropatagium is excluded as a possible surface for launch assistance (in bats, the uropatagium and tail assist take-off—see^33^). This means the broadest surface remaining for propulsion would be webbed feet. The quantitative reconstructions of pterosaur floating postures^1^ confirm that the wings would be in contact with the water (or possibly fully submerged) while floating. Thus, when launching from water, the wings were an important surface area for propulsion.

Water take-off was modelled as a form of modified quadrupedal launch because prior work had suggested that at least some pterosaurs were capable of land-based quadrupedal take-off^4,5^. Among living flyers, water take-off is accomplished through a variety of kinematic options. However, even among birds which are obligate biped launchers on terrestrial substrates, the use of a wing-assisted (or even wing-dominated) quadrupedal launch is not uncommon. Mallard ducks, for example, use the feet and wrists to push off the water surface. In marine pterosaurs like *Anhanguera*, the expanded areas for shoulder extensor muscles and their reduced hindlimbs suggests that water-based quadrupedal take-off was highly likely^6^. Conversely, the low floating posture with the feet far behind the centre of mass^1^ would make bipedal water take-off problematic.

Body mass was estimated from total wingspan following Witton and Naish^29^. For estimates of fluid forces during launch, average seawater density was utilised. To determine the likely speed at which the limbs could be moved on the water, the flapping speed estimates of Pennycuick^34^ were used, which relate wing size, body mass and density of the medium to wing speed. This approach was adapted to pterosaurs by using a folded wingspan and area and varying the degree to which the wing was assumed to be open during the launch. As living water launchers use folded wings with limited points of primary propulsive contact (e.g., quadrupedally launching ducks use their wrists), it is expected that a water-launching pterosaur would only use a limited portion of the wing for propulsion. This would reduce surface tension effects and prevent damage to the thin distal wing spar.

‘Quadrupedal Water Launch Model’ is a tab of the .xlsx spreadsheet in the Supplementary Information S1 that includes all calculations used to estimate water launch performance. Users can manipulate this sheet and input data as they see fit to test alternative models and taxa. Bold values are user inputs; all other values are calculated. The propulsive force for escape is considered to be drag-based and is taken to be:

$${\text{Plate Drag Coefficient}}*\left( {{\text{Foot Area}} + {\text{Folded Wing Area}}} \right) \, *\left( {{\text{Wing Velocity}}^{{2}} } \right) \, *{\text{water density}}*0.{5}$$

Based on the flapping frequency expectations from Pennycuick^34^, flapping frequency varies roughly as body mass to the 3/8 power, gravitational acceleration to the 1/2 power, span to the − 23/24 power, wing area to the − 1/3 power and fluid density to the − 3/8 power.

Wing velocity in the water is estimated using the equation of Pennycuick^34^. While this application is outside the normal range of media over which this relationship is used, we find that it is probably the best estimate of wing speed with variable densities and wing areas.

We present the results for a flight muscle fraction of 0.24 and a hind limb muscle fraction of 0.1. We assume that most of the muscle mass was aerobic, based on specimen size (anaerobic fraction scales positively with body size in flying animals—see^35^). While these are realistic values consistent with the preserved anatomy, we have included all calculations in the ‘Quadrupedal Water Launch Model’ tab of the .xlsx file which allows seeding of any desired body mass, span and muscle fraction values (see Supplementary Information S1).

As counter-motion preloading of the launch muscles is likely limited in water compared to terrestrial launch, we opt to calculate a raw power ratio (i.e., power required/power output, without any preload factor). However, we note that this is quite conservative because some preload was still likely in a water takeoff, especially during the initial escape phase. This is unlikely to have been a major factor in small pterosaurs, but larger individuals may have relied on the preload advantages of burst motions.

## Supplementary Information

Supplementary Information.

## Data Availability

The paper and its accompanying Supplementary Information S1 contain all the data discussed in the study as well as the water launch model.

## Materials availability

Specimen MB.R.3531 is permanently available for academic study by qualified researchers at the collections of the Museum für Naturkunde—Leibniz Institute for Evolution and Biodiversity Science.
